# The Higher the Score, the Darker the Core: The Nonlinear Association Between Grandiose and Vulnerable Narcissism

**DOI:** 10.3389/fpsyg.2018.01305

**Published:** 2018-08-03

**Authors:** Emanuel Jauk, Scott Barry Kaufman

**Affiliations:** ^1^Clinical Psychology and Behavioral Neuroscience, Technische Universität Dresden, Dresden, Germany; ^2^Department of Psychology, University of Graz, Graz, Austria; ^3^Positive Psychology Center, University of Pennsylvania, Philadelphia, PA, United States

**Keywords:** grandiose narcissism, vulnerable narcissism, trifurcated model, nonlinearity, personality functioning

## Abstract

Narcissism is a truly Janusian phenomenon, consisting of both narcissistic grandiosity, exhibitionism, admiration-seeking, boldness, and dominance on the one hand, and narcissistic vulnerability, introversion, withdrawal, hypersensitivity, and anxiety on the other hand. While there is broad consensus that these two seemingly contradictory faces of narcissism can be empirically discerned and have different implications for psychological functioning and mental health, there is not yet agreement on whether grandiose and vulnerable narcissism should be regarded as independent traits or as two manifestations of one personality trait. Previous research indicates that both views hold true when the level of grandiosity is considered a moderating factor: while grandiose and vulnerable narcissism are largely unrelated in the range of normal personality variation, they are correlated in the range of high grandiosity (Jauk et al., 2017b). Here, we replicate and extend this work in an independent sample (*N* = 891) using a more comprehensive narcissism inventory grounded in a new trifurcated model of narcissism. The trifurcated model partitions narcissism into three main personality dimensions: agentic extraversion, antagonism, and neuroticism. We found a significant breakpoint in the association between narcissistic grandiosity and vulnerability at 75% cumulative frequency of grandiosity. While grandiosity and vulnerability are unrelated below this breakpoint (*r* = 0.02), they are strongly correlated above (*r* = 0.45). In the lower range of grandiose narcissism, grandiosity draws more upon agentic extraversion and is largely associated with mental health. In the upper range, however, grandiosity is more strongly linked to antagonism and is substantially associated with fear, negative affect, and depression. These findings provide evidence for the view that grandiose and vulnerable narcissism are distinct traits at lower levels of grandiosity, but blend into an antagonistic core with signs of psychological maladjustment at higher levels. Implications for research on narcissism as a personality trait, as well as clinical practice, are discussed.

## Introduction

Narcissism is a personality trait with two faces: narcissistic grandiosity and vulnerability (Wink, 1991; Dickinson and Pincus, 2003; Pincus and Lukowitsky, 2010; Miller et al., 2011, 2017; Krizan and Herlache, 2018; Kaufman et al., in press). Earlier empirical research on narcissism focused on narcissistic grandiosity, which is the most well-studied characteristic of narcissism and is still dominant in the formal diagnosis of narcissistic personality disorder (American Psychiatric Association, 2013). In the past decade, however, increasing attention has been paid to vulnerable narcissism as an independent trait in narcissism research (e.g., Fossati et al., 2009), and the existence of these two forms of narcissism is now widely recognized (Kaufman et al., in press).

Grandiose narcissism is characterized by self-importance and feelings of superiority, as well as interpersonal exploitativeness (Raskin and Hall, 1981). Vulnerable narcissism, in contrast, is characterized by hypersensitivity, defensiveness, and withdrawal (e.g., Cain et al., 2008). Grandiose and vulnerable narcissism build on distinct nomological networks and are either weakly related or even uncorrelated in the general population (depending on the measures used; Miller et al., 2011, 2013, 2017; see also Jauk et al., 2017b). Thus, these constellations of traits result in seemingly very different personality phenotypes: while those scoring high in grandiose narcissism tend to be extraverted, socially bold, and charming (Back et al., 2010; Dufner et al., 2013; Jauk et al., 2016), those scoring higher in vulnerable narcissism tend to be introverted, anxious, and avoidant (Miller et al., 2012; Hart et al., 2017). Taken together, grandiose and vulnerable narcissism are more related to approach- and avoidance-related behavior, respectively (Spencer et al., 2017).

Yet, both grandiose and vulnerable narcissism share a common dark core consisting of self-centeredness, entitlement, and interpersonal antagonism. In their *trifurcated model* of narcissism, Miller et al. (2016) elucidate the common and differential aspects of grandiose and vulnerable narcissism along Big Five personality traits. Building upon prior work in which narcissism was conceptualized in terms of maladaptive variants of the Five-Factor-Model traits (Glover et al., 2012), Miller et al. proposed a three-factor structure of narcissism consisting of agentic extraversion, antagonism, and neuroticism. Both grandiose and vulnerable narcissism draw strongly upon antagonism at the common core. Beyond this common core, grandiosity is more related to agentic extraversion, whereas vulnerability is more closely linked to neuroticism (Miller et al., 2013, 2016; see also **Figure 2**). The recent trifurcated model is consistent with the also recently proposed narcissism spectrum model (Krizan and Herlache, 2018), and these can be considered the current state of the art in personality research on narcissism (Wright and Edershile, 2017). Here, we will use the trifurcated model as a theoretical basis to investigate differences in the constituent personality factors of grandiose and vulnerable narcissism across the range of narcissistic grandiosity.

### Personality and Clinical Perspectives on Narcissism

While personality researchers frequently emphasize the distinct nature of narcissistic grandiosity and vulnerability, clinical theorists are more inclined to see the common aspects among both (Wright and Edershile, 2017). In their influential review, Pincus and Lukowitsky (2010) argue that individuals diagnosed with narcissistic personality disorder display fluctuating or co-occurring states of narcissistic grandiosity and vulnerability. They assert that “many contemporary clinical experts on narcissistic personality disorder now recognize that grandiose self-states oscillate or co-occur with vulnerable self-states and affective dysregulation” (p. 428). During these states, the clinical presentation of vulnerability can even completely mask narcissistic grandiosity (Pincus et al., 2014). Systematic evidence confirms the notion of oscillating states by showing that individuals identified as grandiose narcissists (following the common DSM-5 criteria) display episodes of heightened vulnerability according to expert ratings (Gore and Widiger, 2016). A similar finding was recently also obtained for lay raters, though with greater emphasis on externalizing symptoms (anger; Hyatt et al., 2017).

The seemingly contradictory views of personality and clinical research on narcissism might be at least partially due to the different populations under study. Insights on the factor and covariance structure among different narcissism aspects are usually obtained from community samples. Per definition, these samples mostly comprise healthy subjects, and, to a lesser extent, individuals with clinically relevant personality constellations at the extremes of the distribution (cf., Carter et al., 2016). Conversely, clinicians commonly deal with severe forms of narcissistic pathology, such as in narcissistic personality disorder, and tend to put emphasis on the common ground of grandiosity and vulnerability (Wright and Edershile, 2017).

### The Nonlinear Association of Grandiose and Vulnerable Narcissism

Following the idea that high trait scores could indicate clinically relevant personality characteristics, we previously attempted to shed light on the complex relationship between narcissistic grandiosity and vulnerability by studying nonlinear associations in a large sample. The analysis revealed that grandiosity and vulnerability are not associated within the normal personality range, but as soon as a critical threshold in the upper range of the grandiose narcissism distribution is met, they are correlated (Jauk et al., 2017b). This finding provided first empirical evidence for the hypothesis that the level of narcissistic grandiosity might moderate its association with narcissistic vulnerability.

The correlation obtained in the higher grandiosity range in the previous study was, however, small. This might not only be attributed to the general difficulty of capturing state-like fluctuations between grandiosity and vulnerability using trait measures (Wright and Edershile, 2017), but also to the specific measures themselves: we previously used the Narcissistic Personality Inventory (NPI; Raskin and Hall, 1979, 1981) and the Hypersensitive Narcissism Scale (HSNS; Hendin and Cheek, 1997) for the assessment of grandiose and vulnerable narcissism. Though these can be considered the long-time standard measures in the non-clinical population, more sophisticated scales are available, and these might be better suited to capture fine-grained variation.

In this study, we thus attempt to replicate and extend the previous work using the aforementioned Five-Factor Narcissism Inventory (FFNI; Glover et al., 2012). The FFNI is capable of assessing grandiose and vulnerable narcissism in a more balanced way than was the case in the previous study. Importantly, the FFNI grandiose and vulnerable scales are intrinsically unrelated (*r* = 0.06; Glover et al., 2012), which allows for a stringent test of the nonlinearity hypothesis.

### Grandiose and Vulnerable Narcissism, Psychological Functioning, and Mental Health

In the general population, grandiose narcissism is positively related to self-esteem (Campbell, 2001, unpublished), different indicators of mental health (e.g., Sedikides et al., 2004), and life satisfaction (Rose, 2002; Egan et al., 2014; Kaufman et al., in press), although most of these associations are due to grandiose narcissism’s association with agentic extraversion (Kaufman et al., in press). Among the core motives, grandiosity is most strongly associated with power and less or negatively associated with affiliation and intimacy (Carroll, 1987; Morf et al., 2011). In line with this, grandiose narcissism is associated with sensitivity to achievement failure, but not to social rejection (Besser and Priel, 2010). This conforms to its dominant-cold position in the interpersonal circumplex (Miller et al., 2012). Concerning psychopathology, grandiosity is mainly linked to externalizing symptoms, such as proactive aggression, and is also correlated with multiple measures of inauthenticity and lack of purpose (Miller et al., 2011; Kaufman et al., in press). Taken together, grandiose narcissism is associated with subjective well-being, and uncorrelated, or even slightly positively associated, with mental health, paired with a dominant interpersonal style. This “happy face” (Rose, 2002) of grandiose narcissism is likely to be driven by its agentic extraversion aspect (Kaufman et al., in press).

Vulnerable narcissism, in contrast, is clearly associated with signs of psychopathology; particularly internalizing symptoms (Kaufman et al., in press). Vulnerable narcissism goes along with negative self-representations (Dickinson and Pincus, 2003); imposter syndrome, a weak sense of self and self-alienation (Kaufman et al., in press); decreased self-esteem (Rose, 2002; Brookes, 2015; Miller et al., 2017); and decreased life satisfaction (Rose, 2002). Vulnerable narcissism is negatively associated with power and affiliation motives (Sturman, 2000), conforming to its low-communion position in the interpersonal circumplex (Miller et al., 2012). Vulnerable narcissism is specifically linked to rejection sensitivity, but not achievement failure (Besser and Priel, 2010). Moreover, vulnerable narcissism is associated with experiencing less positive and more negative affect (Miller et al., 2011, 2017). Consequently, it is accompanied by a broad range of internalizing symptoms, including anxiety and depression (Miller et al., 2011, 2017; Euler et al., 2018; Kaufman et al., in press), and is closely tied to borderline personality features (Miller et al., 2010; Euler et al., 2018).

So far, it can be concluded that – within the range of normal personality variation – narcissistic grandiosity is indicative of egotistic but largely adaptive psychological functioning and mental health. Externalizing symptoms associated with grandiosity are more likely to affect others than the narcissistic individuals themselves (e.g., Miller et al., 2017; Kaufman et al., in press). Vulnerable narcissism, in contrast, is clearly associated with various indicators of maladaptive functioning and mental illness (e.g., in press; Miller et al., 2017).

However, at clinical levels, grandiosity is also associated with poor psychological adjustment: narcissistic personality disorder is substantially comorbid with substance use, bipolar disorder, depression, and eating disorders (Ronningstam, 1996; Stinson et al., 2008). Pathological narcissism, i.e., concurrent grandiosity and vulnerability, is associated with low self-esteem and reduced personality functioning across non-clinical and clinical samples (Pincus et al., 2009) and goes along with numerous symptoms and serious treatment problems in clinical groups (Pincus et al., 2009; Morf et al., 2017).

To explore the validity of the nonlinearity hypothesis with respect to psychological functioning and mental health, we investigate correlates of grandiosity and vulnerability at different levels of grandiosity. Specifically, we examine their relations to motives, fears, self-esteem, affect, and depression. From the literature reviewed above, we predict that grandiosity and vulnerability will display opposing relationships with indicators of mental health in the lower range of grandiosity (Kaufman et al., in press). At high levels, however, we predict that grandiosity will be associated with signs of maladaptive psychological adjustment as well.

### Research Questions and Hypotheses

This study provides a further test of the hypothesis that grandiose and vulnerable narcissism are unrelated within the normal personality variation, but become more closely related at high levels of grandiosity (Jauk et al., 2017b). As in the previous study, we apply segmented regression analysis to detect a possible breakpoint in the correlational structure of the bivariate distribution. Compared to other tests of nonlinearity, segmented regression tests for a discrete breakpoint, which is suitable to detect dose–response relationships (Mueggo, 2003).

We use the FFNI as a measure of narcissism that captures grandiosity and vulnerability in a more balanced fashion. Beyond that, the FFNI allows to shed light on the common and differential involvement of the three factors proposed in the trifurcated model of narcissism (agentic extraversion, antagonism, and neuroticism), along different levels of grandiosity. To elucidate the manifestations of grandiose and vulnerable narcissism in constructs related to psychological functioning and mental health, we incorporated measures of core motives, fears, self-esteem, affect, and depression as external validity measures into the analyses. Also, we administered a short scale of childhood recollections of parental overvaluation; a parenting style that has been associated with grandiose and vulnerable narcissism (Otway and Vignoles, 2006; Brummelmann et al., 2015).

Given that the nonlinearity hypothesis holds true and a breakpoint is detected in the relationship between grandiose and vulnerable narcissism, we make the following predictions:

- Grandiose and vulnerable narcissism will be uncorrelated in the lower range, but correlated in the higher range of grandiosity,
- Within the lower grandiosity range, grandiose narcissism will be associated with agentic extraversion and antagonism, as well as indicators of adaptive psychological functioning and mental health. In contrast, vulnerability will be associated with neuroticism and antagonism, as well as maladaptive functioning and indicators of distress.
- Within the higher grandiosity range, grandiose narcissism will be more strongly associated with antagonism (the potentially most maladaptive characteristic of narcissism). Also, grandiosity will be less strongly associated with indicators of mental health, but more strongly associated with maladaptive characteristics such as fear and depression.

To test for discriminant validity, we also reverse the segmented regression analysis, testing for a change in slope in the relationship between vulnerability and grandiosity as a function of vulnerability. We predict that high vulnerability will be independent from grandiosity at the upper end of vulnerable narcissism, as narcissistic vulnerability was previously found to relate to borderline personality features (Miller et al., 2010; Wright and Edershile, 2017; Euler et al., 2018).

## Materials and Methods

### Participants and Procedure

We aimed to test the nonlinearity hypothesis of grandiose and vulnerable narcissism in a large non-clinical sample (*N* ∼ 1,000, as in Jauk et al., 2017b). The sample size does not only allow for a powerful breakpoint detection, but also ensures that a potential subsample of highly grandiose individuals would be large enough to detect small to medium effects (given the previous estimate of a breakpoint at 90% cumulative frequency, correlations of *r* ≥ 0.19 can be detected in a subsample of *n* = 100). For this, we conducted an online survey via Amazon’s Mechanical Turk.

After exclusion of invalid datasets (see below), the final sample consisted of *N* = 891 English-speaking participants (472 female, 2 were non gender-identified). The mean age of the sample was 37.12 (*SD* = 11.30) years; 56.90% had a bachelor’s degree or higher. There were no missing data in the final sample reported here; individuals who did not complete the online questionnaires or who failed to pass at least one of three attention check items were excluded a-priori (*n* = 320).

The measures included in the study are described below. The constructs of interest were assessed in the following order: childhood recollections of parental overvaluation, depression, narcissism, self-esteem, motives/fears, and positive/negative affect. As this study was part of a larger research project, it also included further individual difference variables that are not analyzed here (these include demographic variables such as ethnicity, religious views, measures of life satisfaction, motivation, grit, interpersonal behavior, pride, selfishness, self-transcendence, communion and communal narcissism, and the Big Five). Participants were instructed to answer all items using the designated response scales (see below). No particular study goal was highlighted in the instruction to participants.

The median time to complete the survey was 36 min (*M* = 49.07 min; *SD* = 45.71 min), and there was no time limit. Participants received monetary compensation of $2 for completion of the study. This study was carried out in accordance with the relevant guidelines and regulations. The protocol was approved by the Ethics committee of the University of Pennsylvania. All subjects gave written informed consent in accordance with the Declaration of Helsinki.

### Measures

#### Narcissism

We assessed grandiose and vulnerable narcissism using the short form of the Five-Factor Narcissism Inventory (FFNI; Glover et al., 2012; Miller et al., 2013; FFNI-SF; Sherman et al., 2015). The FFNI-SF self-report inventory consists of 60 five-point rating scale items (from *1* = *strongly disagree* to *5* = *strongly agree*). The original long form (148 items) and the short form of the FFNI used here were found to display identical factor structures and highly similar correlational profiles to external measures (Sherman et al., 2015). The FFNI was shown to display incremental validity on criteria for narcissistic personality disorder beyond other narcissism measures (Miller et al., 2013).

The FFNI was designed as a comprehensive narcissism measure that builds on maladaptive variants of the Five Factor Model traits. The FFNI was conceptualized in terms of a hierarchical factor structure with 15 facets at the bottom level belonging to five factors at the intermediate level, narcissistic grandiosity, and vulnerability on top level and ultimately a general score of narcissism. More recent factor analytic evidence points to a three-factor structure comprising antagonism, agentic extraversion, and neuroticism (Miller et al., 2016) at the intermediate level (see **Figure 2**). Antagonism relates to both grandiose and vulnerable narcissism. In contrast, grandiosity draws strongly upon agentic extraversion, whereas vulnerability is more closely tied to neuroticism (Miller et al., 2016). In this study, we investigate the linear and nonlinear relations among the higher-order grandiosity/vulnerability model (Glover et al., 2012) and the lower-order trifurcated model (Miller et al., 2016).

Grandiose narcissism was measured as the average of the scales *Indifference, Exhibitionism, Authoritativeness, Grandiose Fantasies, Manipulativeness, Exploitativeness, Entitlement, Lack of Empathy, Arrogance, Acclaim Seeking*, and *Thrill Seeking* (total of 44 items, α = 0.95). Vulnerable narcissism was measured as the average of the scales *Reactive Anger, Shame, Need for Admiration*, and *Distrust* (total of 16 items, α = 0.87). Antagonism was measured as the mean of the subscales *Manipulativeness, Exploitativeness, Entitlement, Lack of Empathy, Arrogance, Reactive Anger, Distrust*, and *Thrill Seeking* (total of 32 items, α = 0.94). Agentic Extraversion was measured as the mean of the scales *Acclaim Seeking, Authoritativeness, Grandiose Fantasies*, and *Exhibitionism* (total of 16 items, α = 0.89). Neuroticism was measured as the mean of the scales *Shame, Indifference* (Reversed), and *Need for Admiration* (total of 12 items, α = 0.89). Detailed descriptions of the subscales and items are given in Glover et al. (2012) or Miller et al. (2013). For the trifurcated model scales, see Miller et al. (2016).

#### Validity Measures

To relate the different narcissism aspects to external validity measures, we assessed motives, fears, self-esteem, affect, and depression. We measured core motives using the Unified Motive Scales (UMS; Schönbrodt and Gerstenberg, 2012). The UMS assesses *power, achievement, affiliation*, and *intimacy* with 10 items each. We used a 5-point rating scale (*1* = *strongly disagree* to *5* = *strongly agree*). We also used the UMS for the assessment of fears. The additional three-item scales allow for measuring fear of *failure, rejection, losing control, losing emotional contact*, and *losing reputation.* Again, we used a 5-point rating scale (*1* = *strongly disagree* to *5* = *strongly agree*). We assessed self-esteem using the Self-Liking/Self-Competence Scale-Revised Version (SLCS-R; Tafarodi and Swann, 2001). The SLCS-R features 16 items rated on a 5-point scale (*1* = *strongly disagree* to *5* = *strongly agree*). Because the dimensions self-worth and self-competence were both highly associated with the total score (*r*s = 0.96, 0.87) and did not yield different results in the analyses reported below, we report only results for overall self-esteem in the following. Positive and negative affect were measured using the PERMA profiler (Butler and Kern, 2015). The PERMA profiler assesses positive and negative affect with three items each, which are rated on an 11-point scale (*0* to *10* using different anchors). We assessed depression using the 20-item Center for Epidemiological Studies Depression scale (CES-D; Radloff, 1977). The scale uses a 4-point rating scale (form *0* to *3*) based on the frequency of the occurrence of depressive symptoms. Finally, we administered five self-constructed items (scored on a 5-point scale (from *1* = strongly disagree to *5* = strongly agree, α = 0.86) to assess childhood recollections of parental overvaluation; a candidate childhood antecedent of narcissism (Brummelmann et al., 2015). Example items for parental overvaluation are as follows: “As a child, I was constantly told that I was ‘destined for greatness”’ or “As a child, I was constantly told that I would be a great success in the eyes of the world.”

### Data-Analytical Strategy

The main aim of this study was to investigate linear and nonlinear associations among narcissistic grandiosity and vulnerability, the trifurcated model of narcissism, as well as validity measures. For this, we first investigated associations among narcissism aspects as well as validity measures in the full sample.

Next, we applied segmented regression analysis to test a possible nonlinear association between narcissistic grandiosity and vulnerability. Segmented regression is an iterative computational procedure capable of detecting a breakpoint (i.e., change in slope) between two continuous variables (Ulm, 1991; Haybach and Küchenhoff, 1997). It is commonly used in epidemiology, for instance, to investigate dose-response research questions such as at which breakpoint ψ does a stressor *X* have an impact on health outcome *Y*? We previously used segmented regression to determine the breakpoint in grandiose narcissism (*X*) upon which grandiosity is correlated with vulnerability (*Y*; Jauk et al., 2017b); the method reported here exactly corresponds to this study.

Precursor analyses to segmented regression include tests for influential data points and nonlinearity in the data. We tested for outliers in the bivariate distribution by means of Mahalanobis distance including the FFNI variables grandiose and vulnerable narcissism. The analysis did not yield evidence for influential data points; all distances were well below the critical value of χ^2^(2) = 13.82. To determine nonlinearity of the data, we used a quadratic over a linear predictor term in a standard regression model (cf. Jauk et al., 2013). At this, we avoided collinearity of the predictors by means of residual centering (Lance, 1988). Again, the method corresponds exactly to our previous study (Jauk et al., 2017b). The quadratic predictor term explained significant incremental amount of variance in vulnerable narcissism (Δ*R*^2^ = 0.03, *p* < 0.001), which indicates significant nonlinearity in the data above and beyond a linear effect (β_linear_ = 0.21, *p* < 0.001; β_quadratic_ = 0.16, *p* < 0.001).

We estimated the segmented regression model using the *segmented* package (Mueggo, 2008) for the open statistics software R (R 3.4.3, RStudio Version 1.1.419, segmented package 0.5–3.0). FFNI grandiose narcissism served as the independent (*X*), FFNI vulnerable narcissism as the dependent (*Y*) variable. The algorithm has to be supplied with an arbitrary initial guess parameter for the breakpoint. We used an initial guess parameter of ψ_0_ = 2.5 points in the FFNI grandiose narcissism raw score^1^ . Significance of the empirically determined breakpoint was tested using the Davies test (Davies, 1987). The Davies test estimates the probability of a significant change in slope (H1) under the assumption that the breakpoint parameter ψ is non-existent under H0. The test needs to be supplied with a number of *K* equally spaced evaluation points between the 5 and 95% quantiles of the independent variable. According to common recommendations, this parameter was set to *K* = 7 (Mueggo, 2008). Significance testing was performed two-tailed at α = 0.05.

As a significant breakpoint was detected (see below), we next investigated the associations between grandiose and vulnerable narcissism, their constituent components in terms of the trifurcated model, and validity measures in subsamples below and above that breakpoint. Correlations below and above the breakpoint were tested for statistically significant differences using Steiger’s *z*-test for comparison of two independent correlations (corrected for multiple testing; see below). For visualization purposes, we additionally present plots depicting the strength of correlations along the grandiose narcissism dimension. To investigate which factors of the trifurcated model account for the common variance among grandiose and vulnerable narcissism, we also report partial correlation analyses. Also, we report differences in mean structure, as the overall levels might be relevant to the interpretation of correlation differences^2^.

Lastly, to investigate the specificity of the obtained findings, we reversed the segmented regression analysis, with vulnerable narcissism being the predictor (*X*), and grandiose narcissism being the criterion (*Y*) variable. We hypothesized that vulnerable narcissism would be independent from grandiose narcissism at high levels of vulnerable narcissism.

## Results

### Descriptive Statistics and Correlations (Full Sample)

**Table 1** (p. 7) shows the mean and correlation structure of the variables under study in the full sample. Grandiose and vulnerable narcissism are moderately correlated throughout the whole range, and both are highly associated with antagonism. Consistent with prior research, grandiosity is more tied to agentic extraversion, and vulnerability is more closely linked to neuroticism.

**Table 1:** Mean and correlation structure of narcissism aspects and validity measures in the full sample and lower/higher grandiosity subsamples.

	Full sample (*N* = 891)																			Lower grandiosity subsample (*n* = 665)			Higher grandiosity subsample (*n* = 226)					
	*M* (*SD*)	1	2	3	4	5	6	7	8	9	10	11	12	13	14	15	16	17	18	*M* (*SD*)	1	2	*M* (*SD*)	1	2	*p* Δ *M*	*p* Δ *r*1,x	*p* Δ *r*2,x
**G/V-model**																												
Grandiose narcissism (1)	2.46 (0.69)																			2.14 (0.44)			3.40 (0.35)					
Vulnerable narcissism (2)	2.84 (0.78)	0.21																		2.75 (0.81)	0.02		3.10 (0.59)	0.45		**<0.001**	**<0.001**	
**Trifurcated model**																												
Antagonism (3)	2.25 (0.72)	0.88	0.53																	1.94 (0.48)	0.69	0.57	3.17 (0.47)	0.83	0.71	**<0.001**	**<0.001**	**0.002**
Agentic extraversion (4)	2.88 (0.83)	0.84	0.10	0.56																2.59 (0.71)	0.79	-0.03	3.73 (0.48)	0.51	0.10	**<0.001**	**<0.001**	0.092
Neuroticism (5)	2.99 (0.90)	-0.24	0.75	0.02	-0.14															3.06 (0.97)	-0.26	0.81	2.76 (0.62)	0.08	0.74	**<0.001**	**<0.001**	0.023
**Motives**																												
Power (6)	2.65 (0.96)	0.71	0.05	0.52	0.77	-0.19														2.35 (0.88)	0.63	-0.07	3.52 (0.58)	0.21	-0.03	**<0.001**	**<0.001**	0.604
Achievement (7)	3.88 (0.75)	0.32	-0.17	0.07	0.51	-0.24	0.46													3.80 (0.79)	0.30	-0.24	4.13 (0.57)	0.25	-0.10	**<0.001**	0.485	0.062
Affiliation (8)	2.87 (0.99)	0.40	-0.18	0.16	0.55	-0.13	0.42	0.36												2.71 (0.99)	0.30	-0.31	3.36 (0.81)	0.27	0.08	**<0.001**	0.673	**<0.001**
Intimacy (9)	3.76 (0.81)	0.05	-0.03	-0.08	0.24	0.05	0.15	0.33	0.40											3.76 (0.85)	0.05	-0.06	3.76 (0.69)	0.24	0.16	0.999	0.012	0.004
**Fears**																												
Failure (10)	3.29 (1.10)	-0.08	0.61	0.13	-0.10	0.62	-0.15	-0.24	-0.17	0.10										3.31 (1.14)	-0.17	0.65	3.22 (1.00)	0.22	0.57	0.257	**<0.001**	0.099
Rejection (11)	3.03 (1.15)	-0.21	0.59	0.01	-0.16	0.77	-0.18	-0.26	-0.15	0.03	0.61									3.11 (1.21)	-0.24	0.65	2.79 (0.91)	0.08	0.49	**<0.001**	**<0.001**	**0.002**
Losing control (12)	3.03 (1.07)	0.09	0.67	0.29	0.06	0.60	0.06	-0.12	-0.10	0.11	0.70	0.60								2.99 (1.10)	-0.01	0.68	3.16 (0.98)	0.32	0.63	0.029	**<0.001**	0.257
Losing contact (13)	3.26 (1.08)	-0.05	0.52	0.09	0.02	0.64	-0.05	-0.10	0.09	0.32	0.59	0.58	0.59							3.28 (1.12)	-0.12	0.54	3.22 (0.98)	0.27	0.54	0.428	**<0.001**	1.00
Losing reputation (14)	3.28 (1.10)	0.01	0.30	0.05	0.12	0.40	0.09	0.10	0.09	0.20	0.37	0.33	0.40	0.46						3.29 (1.14)	-0.02	0.29	3.25 (0.98)	0.27	0.41	0.657	**<0.001**	0.077
**Self-Esteem** (15)	3.43 (0.79)	0.26	-0.58	-0.03	0.35	-0.65	0.34	0.50	0.36	0.24	-0.54	-0.58	-0.49	-0.39	-0.07					3.35 (0.84)	0.25	-0.67	3.65 (0.54)	-0.01	-0.44	**<0.001**	**<0.001**	**<0.001**
**Affect**																												
Positive (16)	6.60 (2.29)	0.18	-0.43	-0.08	0.30	-0.39	0.21	0.39	0.47	0.45	-0.32	-0.35	-0.30	-0.12	0.00	0.69				6.45 (2.39)	0.13	-0.54	7.04 (1.92)	0.27	-0.09	**<0.001**	0.059	**<0.001**
Negative (17)	3.91 (2.23)	0.10	0.66	0.34	-0.04	0.51	-0.07	-0.24	-0.18	-0.11	0.52	0.44	0.56	0.38	0.16	-0.63	-0.57			3.77 (2.16)	-0.07	0.67	4.34 (2.37)	0.34	0.61	**0.001**	**<0.001**	0.188
**Depression** (18)	1.91 (0.64)	0.13	0.57	0.37	-0.05	0.42	-0.08	-0.24	-0.23	-0.22	0.41	0.36	0.44	0.27	0.08	-0.63	-0.66	0.77		1.86 (0.63)	-0.05	0.58	2.06 (0.66)	0.33	0.52	**<0.001**	**<0.001**	0.266
**Parental overvaluation** (19)	2.99 (1.03)	0.39	0.06	0.28	0.42	-0.01	0.31	0.19	0.39	0.23	0.07	0.01	0.11	0.13	0.18	0.19	0.27	-0.01	-0.06	2.81 (1.00)	0.26	-0.06	3.51 (0.92)	0.31	0.24	**<0.001**	**<0.001**	**<0.001**

*Full sample: Correlations above r = 0.06 are significant at p < 0.05, correlations above r = 0.08 are significant at p < 0.01. Lower grandiosity subsample: Correlations above r = 0.08 are significant at p < 0.05, correlations above r = 0.10 are significant at p < 0.01. Higher grandiosity subsample: Correlations above r = 0.13 are significant at p < 0.05, correlations above r = 0.17 are significant at p < 0.01. The last three columns show significance of differences between means (p Δ M) and correlations (grandiosity [p Δ r1,x] and vulnerability [p Δ r2,x]). Significant differences after correction for multiple testing are printed in bold. Bonferroni correction was performed for the number of comparisons within each family of tests (i.e., comparisons within each column) for the last three columns.*

Narcissistic grandiosity is accompanied by motives for power, achievement, and affiliation, but not intimacy. Vulnerability displays small negative associations with affiliation and intimacy, but shows no correlation with the other motives. Concerning fears, grandiosity displays small and mostly negative associations, especially with fear of rejection. Vulnerability displays high positive correlations with all dimensions of fear, providing further support that vulnerable narcissism is more tied to a general avoidance motivation than grandiose narcissism.

Grandiose narcissism is moderately positively associated with self-esteem, whereas vulnerable narcissism is strongly negatively associated with self-esteem. Grandiosity is not markedly associated with experiencing positive and negative affect or depression, whereas vulnerability goes along with substantially reduced positive and increased negative affect, as well as depression. Lastly, grandiosity displays a substantial correlation with parental overvaluation, while vulnerability was uncorrelated with parental overvaluation.

### Segmented Regression Model

**FIGURE 1:**
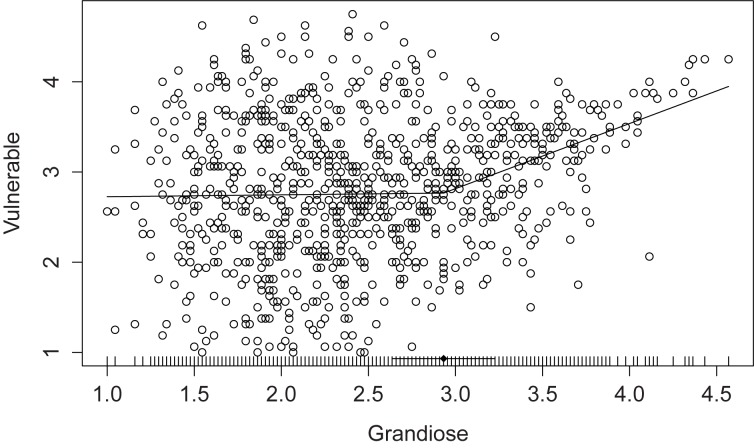
Segmented regression model of the relationship between grandiose and vulnerable narcissism within the Five-Factor Narcissism Inventory (FFNI-SF). Horizontal line indicates 95% *CI* of the breakpoint.

To test for a possible breakpoint in the association between narcissistic grandiosity and vulnerability, we applied segmented regression analysis. The breakpoint was estimated at an FFNI grandiosity raw score of 2.93, corresponding to 75.50% cumulative frequency. This breakpoint was statistically significant according to the Davies test for a change in slope (*p* < 0.001). **Figure 1** depicts the segmented regression model. The correlations between narcissistic grandiosity and vulnerability were *r*_grand<2.93_ = 0.02 (*p* = 0.66, *n* = 665) and *r*_grand>2.93_ = 0.45 (*p* < 0.001, *n* = 226) below and above the breakpoint, respectively. These correlations differ significantly according to Steiger’s *z*-test (*z* = 6.00, *p* < 0.001).

### Descriptive Statistics and Correlations (Lower vs. Higher Grandiosity Subsamples)

**Table 1** (p. 8) displays the mean structure and associations between grandiose and vulnerable narcissism, the trifurcated model of narcissism, and validity measures in the lower versus higher grandiosity subsamples. Again, we tested for significant differences in the correlation structure of both subsamples using Steiger’s *z*-test. As a regular *p* of 0.05 appeared too liberal in the light of the large sample (high statistical power) and the many tests, we applied criterion-wise Bonferroni correction (i.e., correction for multiple testing for each family of tests sharing one variable, or by the number of comparisons in each of the last three columns in **Table 1**, p. 8). Significant differences after correction are marked in bold.

In the higher as compared to the lower grandiosity subsample, grandiose narcissism was more strongly related to antagonism and less related to agentic extraversion (though the mean of agentic extraversion was higher in the higher subsample). The negative correlation with neuroticism in the lower subsample vanished in the upper subsample, resulting in a near-zero correlation (the mean level of neuroticism was lower in the higher as compared to the lower subsample). As supplemental information, Supplementary Table A1 additionally displays differences in mean and correlation structure for all 15 FFNI-SF facets.

To test which of the trifurcated model factors account for the common variance among grandiose and vulnerable narcissism in the higher grandiosity subsample, we additionally computed partial correlations between both, controlling for the factors of the trifurcated model. Controlling for agentic extraversion did not alter the correlation substantially (*r*_GV.AE_ = 0.47). Controlling for neuroticism mildly increased the correlation (*r*_GV.N_ = 0.57). Controlling for antagonism, finally, yielded a negative correlation (*r*_GV.A_ = -0.35). This indicates that, assuming there were no differences in antagonism, grandiosity would be accompanied by *decreased* vulnerability. From this, we can conclude that antagonism accounts for the positive association between grandiosity and vulnerability at high levels of grandiosity.

Grandiosity was less associated with the power motive in the higher than the lower subsample (though, note, that the mean was higher in the higher subsample). There was a slight effect for an association with intimacy in the higher subsample, but the difference was not significant after Bonferroni correction. Grandiosity was accompanied by a higher proneness to experiencing fear of failure, fear of rejection, fear of losing control, fear of losing emotional contact, and fear of losing reputation in the higher subsample (though the absolute correlation for fear of rejection was not significant). The means between groups did not differ (except for fear of rejection). Grandiosity was only associated with self-esteem in the lower, but not in the higher subsample, and this difference was statistically significant (overall self-esteem was higher in the higher subsample). Grandiosity was related to experiencing negative affect and depression in the higher subsample, but not in the lower. Grandiosity was also substantially related to experiencing positive affect in the higher subsample, though the difference between correlations was not significant. The means of positive affect, negative affect, and depression were higher in the higher than in the lower grandiosity subsample.

Concerning the correlates of narcissistic vulnerability in the higher vs. lower grandiosity subsample, the association with antagonism increased, but there were no significant differences in the correlations with agentic extraversion or neuroticism. The negative correlation with self-esteem was less pronounced in the higher subsample, but still substantial. The correlation with experiencing positive affect changed from negative to zero in the lower vs. higher subsample, as did the correlation with the affiliation motive. Notably, in the higher grandiosity subsample, vulnerability was positively associated with parental overvaluation. None of the other effects differed significantly.

### Discriminant Validity Analysis

To investigate the specificity of the obtained findings, we reversed the segmented regression analysis. We tested for a change in slope in the relationship between narcissistic vulnerability (*X*) and grandiosity (*Y*) as a function of the level of vulnerability. A breakpoint was estimated at an FFNI vulnerability raw score of 3.50, corresponding to 80.00% cumulative frequency. The correlations with grandiosity below and above this breakpoint were *r*_vuln<3.50_ = 0.30 (*p* < 0.001, *n* = 713) and *r*_vuln>3.50_ = -0.02 (*p* = 0.79, *n* = 178). These coefficients are significantly different according to Steiger’s *z*-test (*z* = 3.90, *p* < 0.001). This indicates that, contrary to high grandiosity being associated with vulnerability, high vulnerability is independent from grandiosity.

## Discussion

This study provides a further test of the hypothesis of a nonlinear association between grandiose and vulnerable narcissism (Jauk et al., 2017b). We expected that grandiose and vulnerable narcissism would not be correlated in the lower range of grandiosity, but should be related to each other at high levels of grandiosity. This hypothesis was confirmed by segmented regression analysis: a significant breakpoint in the bivariate relationship was detected at 75% cumulative frequency of narcissistic grandiosity (see **Figure 1**). While the two forms of narcissism were unrelated below the breakpoint, they were substantially associated above it. In the following, we will first discuss the general implications of this finding. We will then highlight the differential relations of grandiosity and vulnerability to the factors of the trifurcated model of narcissism, and finally turn to the discussion of differential relationships with indicators of psychological functioning and mental health.

### Further Evidence for the Nonlinear Association of Grandiose and Vulnerable Narcissism

The first aim of this study was to replicate the findings obtained by Jauk et al. (2017b) in an independent sample, using the FFNI-SF as a comprehensive measure of grandiose and vulnerable narcissism. Data analyses confirmed our hypothesis of a nonlinear association, as we observed a significant breakpoint (at 75% cumulative frequency of grandiosity) in the relationship between both forms of narcissism. Grandiosity and vulnerability were uncorrelated (*r* = 0.02) below the breakpoint, and were substantially correlated (*r* = 0.45) above the breakpoint. The difference between both was highly statistically significant. Notably, the difference between correlations was more pronounced than in the previous study (*r*s = -0.09/0.20; Jauk et al., 2017b). This is mostly likely due to differences among the scales: while the previous study employed the NPI and the HSNS as trait measures of grandiose and vulnerable (hypersensitive) narcissism, we used the more comprehensive FFNI-SF in this study. The FFNI-SF allows for a more reliable and balanced assessment of both grandiose and vulnerable narcissism and can capture more fine-grained variation among both vulnerable and grandiose forms of narcissism.

Our findings indicate that grandiose and vulnerable narcissism are indeed two distinct faces of narcissism within the general population, conforming to current structural models of narcissism in personality research (Miller et al., 2016; Krizan and Herlache, 2018). In the upper range of grandiosity, however, grandiosity and vulnerability are substantially related, as posited by current clinical models of narcissism (Pincus and Lukowitsky, 2010). The correlation between grandiosity and vulnerability obtained here for the upper range of grandiosity is similar to what is found in the literature on pathological narcissism (Wright et al., 2010). The findings obtained here might thus help to bridge the gap between personality and clinical accounts of narcissism; a phenomenon at the crossroads between both research traditions (Cain et al., 2008). Based on the present and previous findings (Jauk et al., 2017b), we argue that both personality and clinical views on narcissism hold true. Which model actually applies depends on the level of narcissistic grandiosity used as a moderator.

### The Structure of Narcissism Along the Range of Grandiosity

**FIGURE 2:**
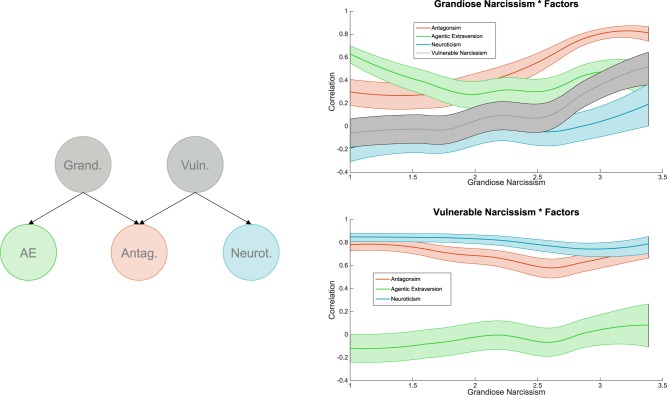
Left: Schematic illustration of the trifurcated model of narcissism (adapted from Miller et al., 2016). AE, agentic extraversion. Right: Visualization of the correlation between grandiose (top panel) and vulnerable (bottom panel) narcissism and the factors of the trifurcated model as a function of grandiose narcissism. Plots display the correlation (including 95% *CI* band) in windows of *n* = 250 data points, iteratively computed for every *X*-value (starting from the lowest) and smoothed using a loess filter. The upper tail uses a minimum of *n* = 100 data points as ceiling.

To gain a more profound understanding of the lower-level personality factors constituting narcissistic grandiosity and vulnerability at different levels of grandiosity, we adopted the framework of the trifurcated model of narcissism. Results show that, at lower levels of grandiosity, grandiose narcissism is strongly fueled by agentic extraversion (*r* = 0.79), followed by antagonism (*r* = 0.69) and is negatively associated with neuroticism (*r* = -0.29; see **Figure 2**, top panel). This pattern is in line with previous findings on the structure of grandiose narcissism (Miller et al., 2016) and points to grandiose narcissism as a blend of extraverted and emotionally stable characteristics with an antagonistic core. On the contrary, narcissistic vulnerability was strongly associated with neuroticism (*r* = 0.81) and, to a lesser extent, antagonism (*r* = 0.57), but not with agentic extraversion (*r* = -0.03; see **Figure 2**, bottom panel), which is also in line with previous findings (Miller et al., 2016).

The picture looked differently in the higher subsample of the grandiose narcissism distribution: Grandiose narcissism was now mainly driven by antagonism (*r* = 0.83) and, only to a lesser extent, by extraversion (*r* = 0.51; see **Figure 2**, top panel). The association among grandiosity and emotional stability vanished in the higher subsample (*r* = 0.08; see **Figure 2**, top panel). Vulnerable narcissism, on the contrary, showed similar correlates in the lower and higher grandiosity subsamples (see **Figure 2**, bottom panel). However, the increase of antagonism alongside vulnerability was statistically significant (see **Table 1**), further underpinning the notion of an increasingly antagonistic core at higher levels of grandiosity.

Taken together, we conclude that grandiose and vulnerable narcissism become more closely intertwined at high levels of grandiosity, which is reflected in a strong antagonistic core toward the upper end of the grandiosity distribution. This is confirmed by the partial correlation analysis indicating that antagonism can fully account for the common variance among grandiosity and vulnerability at high levels of grandiosity. More adaptive and protective aspects, such as agentic extraversion, are less closely related to grandiosity (though the mean was higher), allowing the narcissism to become more “unleashed,” so to speak. The highly grandiose narcissistic person might thus be characterized by a markedly antagonistic interpersonal style, along vulnerable aspects, such as need for admiration (Glover et al., 2012). In more casual words: While moderate grandiosity might point to effective and charismatic leaders, high grandiosity might describe individuals being entitled and needy.

### Psychological Functioning and Mental Health in Lower vs. Higher Grandiosity Subsamples

Our data further show that, in the lower grandiosity subsample, grandiose narcissism seems to be largely associated with adaptive, though egotistical, psychological functioning, and mental health. Grandiosity was positively associated with self-esteem and the power motive, while being negatively associated with fear of rejection and fear of failure. This picture largely conforms to the “happy face” of narcissism (Rose, 2002). In the higher grandiosity subsample, in contrast, grandiose narcissism was not associated with self-esteem anymore, and the correlation with the power motive was markedly reduced (though, note, that the means of self-esteem and power were still higher). Instead, grandiose narcissism was associated with almost all of the fears measured in this study, particularly the fear of losing control. Grandiose narcissism was further substantially associated with depression (and a higher mean level of depression). Interestingly, not only grandiosity, but also vulnerability was associated with childhood recollections of parental overvaluation in the higher grandiosity subsample. This result is in line with those of Otway and Vignoles (2006) and points to a common developmental mechanism of both aspects of narcissism among highly grandiose individuals.

Taken together, the pattern of results suggests that, contrary to the “happy face” of narcissism at moderate levels of grandiosity, high levels of grandiosity are accompanied by indicators of psychological maladjustment and mental illness. This is especially evident in the substantial correlations among grandiose narcissism and negative affect, depression, as well as fear of losing control. These results are more in line with clinical than personality research on narcissistic grandiosity (Ronningstam, 1996; Stinson et al., 2008; Pincus and Lukowitsky, 2010) and highlight the importance of considering the overall level of grandiosity as a moderating factor in correlational research.

Interestingly, grandiose narcissism seems to be more related to experiencing both positive *and* negative affect at higher levels of grandiosity (thought the correlational difference of the former is not statistically significant, the correlation in the upper range is substantial). Taken together with the increased fear of losing control, this pattern seems to point into the direction of greater general affective dysregulation among higher levels of grandiose narcissism (cf. Wright and Edershile, 2017). Tentatively speaking, the higher extremity of affective states might indicate an experiential and behavioral pattern of “living on the edge,” in which experiencing higher positive affect might give space to experiencing higher negative affect, and vice versa. This pattern might also link narcissism to other personality disorders, particularly histrionic (Miller et al., 2011). However, this supposition needs to be tested in further studies.

The findings reported here might have important implications not only for personality researchers but also for clinicians. The observation that narcissistic vulnerability increases at high levels of grandiosity means that clinicians should be particularly attentive to signs of narcissistic vulnerability in clients presenting as highly grandiose. Importantly, narcissistic vulnerability need not necessarily be overtly expressed (Pincus and Lukowitsky, 2010), but can have dramatic implications for psychotherapy, which is most evident in suicidal ideation and behavior (Pincus et al., 2009). However, though our findings indicate a higher likelihood of vulnerability at high levels of grandiosity, they should not be mistaken as a cut-off point. Clinical diagnoses of narcissism should be based on the particular situation of the single individual, and their level of adaptive functioning (Pincus et al., 2014).

### Limitations and Prospects

The findings reported here might be subject to controversy, as they could be seen as supporting a mask model of narcissism; i.e., the psychodynamically inspired notion that narcissistic grandiosity is a facade to mask an underlying vulnerable self (Kernberg, 1975; Kohut, 1977). Though there is some recent neurophysiological evidence in favor of this model (Cascio et al., 2015; Jauk et al., 2017a), there is also strong behavioral evidence against it (Campbell et al., 2007; Marissen et al., 2016). The data reported here cannot speak to the causal nature of grandiosity and vulnerability. However, the direction of causality commonly implicated in the mask model (vulnerability leads to grandiosity) is not the only possible one. Another plausible interpretation of the current findings would be that high grandiosity leads to vulnerability, rather than vice versa. As argued above, the data reported here might indicate a pattern of pronounced ups and downs in affective experience among anyone who possesses the strong core of narcissism (e.g., excessive self-focus, entitlement, interpersonal antagonism).

A popular argument against the view that high grandiosity goes along with vulnerable aspects is the common observation that there are in fact individuals with apparently exaggerated narcissistic grandiosity, who are highly successful in vocational and/or social contexts and do not appear to show even the slightest signs of vulnerability. Our data do not stand at odds with such observations, as our findings only imply a higher likelihood of vulnerability at high levels of grandiosity. The other way around, our additional discriminant validity analysis shows that high vulnerability is not dependent on grandiosity. The factors that determine whether someone actually displays increased vulnerability alongside high grandiosity might be an interesting topic for future studies.

The findings reported here might also have implications for the understanding of the Dark Triad of personality (narcissism, Machiavellianism, and psychopathy), while narcissism is commonly viewed as the “lightest” among the Dark Triad traits (e.g., Rauthmann and Kolar, 2012; see also Furnham et al., 2013), our findings indicate that high grandiosity is accompanied by diverse maladaptive characteristics and a strong antagonistic core. Given that interpersonal antagonism also constitutes the core of the Dark Triad (Jones and Figueredo, 2013), future studies could investigate whether the common core of the Dark Triad is also stronger at higher trait levels of narcissism.

From a methodological point of view, it needs to be acknowledged that some of the correlation and mean differences in the relationship between grandiosity and other variables reported here are also accompanied by differences in variance (i.e., heteroscedastic relationships). For instance, while the correlation between grandiosity and agentic extraversion decreases in the higher grandiosity subsample, the mean of agentic extraversion is still higher in the higher than in the lower grandiosity subsample. This indicates a bivariate relationship that levels off at high grandiosity (i.e., increases in grandiosity are associated with increases in extraversion to a lesser extent), which might at least partially be due to restricted variance in extraversion at higher levels of grandiosity. The effects for extraversion, the power motive, and self-esteem reported in this study should thus be interpreted with care. However, variance restriction is less likely to produce higher correlations toward the end of the distribution, which is why this issue does not concern our main result of higher vulnerability along high grandiosity. Nonetheless, all of the findings reported here await further replication in independent datasets. To evaluate the viability of the nonlinearity hypothesis, it seems particularly important to report not only positive findings but also null findings. From the results of this and our previous study (Jauk et al., 2017b), we suggest that a sample split at +1 *SD* above the mean (which is in the confidence interval of both studies) might be a reasonable approximation for a simple test of the nonlinearity hypothesis without the use of segmented regression.

## Conclusion

This study set out to test the nonlinearity hypothesis of the relationship between grandiose and vulnerable narcissism. Data analysis confirmed this hypothesis, as it showed a significant breakpoint in the bivariate distribution at 75% cumulative frequency of grandiosity. While grandiosity and vulnerability are unrelated below this breakpoint, they are substantially related at high levels of grandiosity. Grandiosity is driven more by agentic extraversion in the lower range, whereas grandiosity and vulnerability blend into an antagonistic core in the higher range of grandiosity. While grandiosity is indicative of egotistic but adaptive psychological functioning in the lower range, it is associated with signs of affective dysregulation and maladaptive functioning in the higher range. We believe these findings have important implications for both researchers who investigate narcissism from a personality perspective, as well as clinicians who are attempting to help those displaying core narcissistic features.

## Author Contributions

EJ and SBK developed the study concept, performed the data analysis, and provided interpretation of the results. SBK designed the study, performed the testing and data collection, and provided critical revisions. EJ drafted the manuscript. Both authors approved the final version of the manuscript for submission.

## Conflict of Interest Statement

The authors declare that the research was conducted in the absence of any commercial or financial relationships that could be construed as a potential conflict of interest.

## Footnotes

1 This initial guess parameter corresponds to the scale average. Different initial guess parameters lead to the same results.

2 While the correlations inform about mean differences within groups, these overall tests inform about mean differences between groups.
