# Supplementary material for: The Higher the Score, the Darker the Core: The Nonlinear Association Between Grandiose and Vulnerable Narcissism
Source: Front Psychol. 2018 Aug 3;9:1305. doi: 10.3389/fpsyg.2018.01305 (PMC6088174; doi:10.3389/fpsyg.2018.01305)
Supplement: Supplementary file 1 [file Table_1.DOCX]

Supplemental Table A1. Mean and correlation structure of FFNI-SF narcissism factors and subscales in the full sample and lower/higher grandiosity subsamples.

|  | Full sample (*N* = 891) | | | | | | | | | | | | | | | | | | |  |  | Lower Grandiosity Subsample  (*n* = 665) | | |  | Higher Grandiosity Subsample  (*n* = 226) | | |  |  |  |  |
| --- | --- | --- | --- | --- | --- | --- | --- | --- | --- | --- | --- | --- | --- | --- | --- | --- | --- | --- | --- | --- | --- | --- | --- | --- | --- | --- | --- | --- | --- | --- | --- | --- |
|  | *M* (*SD*) | 1 | 2 | 3 | 4 | 5 | 6 | 7 | 8 | 9 | 10 | 11 | 12 | 13 | 14 | 15 | 16 | 17 | 18 | 19 |  | *M* (*SD*) | 1 | 2 |  | *M* (*SD*) | 1 | 2 |  | *p* Δ *M* | *p* Δ *r*1,x | *p* Δ *r*2,x |
| G/V-model |  |  |  |  |  |  |  |  |  |  |  |  |  |  |  |  |  |  |  |  |  |  |  |  |  |  |  |  |  |  |  |  |
| Grandiose Narcissism (1) | 2.46 (0.69) |  |  |  |  |  |  |  |  |  |  |  |  |  |  |  |  |  |  |  |  | 2.14 (0.44) |  |  |  | 3.40 (0.35) |  |  |  |  |  |  |
| Vulnerable Narcissism (2) | 2.84 (0.78) | .21 |  |  |  |  |  |  |  |  |  |  |  |  |  |  |  |  |  |  |  | 2.75 (0.81) | .02 |  |  | 3.10 (0.59) | .45 |  |  | **<.001** | **<.001** |  |
|  |  |  |  |  |  |  |  |  |  |  |  |  |  |  |  |  |  |  |  |  |  |  |  |  |  |  |  |  |  |  |  |  |
| Trifurcated model |  |  |  |  |  |  |  |  |  |  |  |  |  |  |  |  |  |  |  |  |  |  |  |  |  |  |  |  |  |  |  |  |
| Antagonism (3) | 2.25 (0.72) | .88 | .53 |  |  |  |  |  |  |  |  |  |  |  |  |  |  |  |  |  |  | 1.94 (0.48) | .69 | .57 |  | 3.17 (0.47) | .83 | .71 |  | **<.001** | **<.001** | **.002** |
| Agentic Extraversion (4) | 2.88 (0.83) | .84 | .10 | .56 |  |  |  |  |  |  |  |  |  |  |  |  |  |  |  |  |  | 2.59 (0.71) | .79 | -.03 |  | 3.73 (0.48) | .51 | .10 |  | **<.001** | **<.001** | .092 |
| Neuroticism (5) | 2.99 (0.90) | -.24 | .75 | .02 | -.14 |  |  |  |  |  |  |  |  |  |  |  |  |  |  |  |  | 3.06 (0.97) | -.26 | .81 |  | 2.76 (0.62) | .08 | .74 |  | **<.001** | **<.001** | .023 |
|  |  |  |  |  |  |  |  |  |  |  |  |  |  |  |  |  |  |  |  |  |  |  |  |  |  |  |  |  |  |  |  |  |
| Subscales |  |  |  |  |  |  |  |  |  |  |  |  |  |  |  |  |  |  |  |  |  |  |  |  |  |  |  |  |  |  |  |  |
| Reactive Anger (6) | 2.50 (1.07) | .48 | .75 | .69 | .35 | .33 |  |  |  |  |  |  |  |  |  |  |  |  |  |  |  | 2.27 (1.02) | .31 | .73 |  | 3.18 (0.92) | .44 | .83 |  | **<.001** | .050 | **.001** |
| Shame (7) | 3.34 (1.12) | -.16 | .77 | .07 | -.11 | .88 | .36 |  |  |  |  |  |  |  |  |  |  |  |  |  |  | 3.40 (1.18) | -.22 | .81 |  | 3.14 (0.9) | .28 | .80 |  | **.001** | **<.001** | .714 |
| Indifference (8) | 2.94 (1.15) | .47 | -.31 | .30 | .23 | -.81 | -.02 | -.54 |  |  |  |  |  |  |  |  |  |  |  |  |  | 2.71 (1.16) | .33 | -.47 |  | 3.62 (0.82) | .43 | -.07 |  | **.001** | .131 | **<.001** |
| Need for Admiration (9) | 2.57 (0.97) | .06 | .82 | .32 | .01 | .82 | .47 | .67 | -.45 |  |  |  |  |  |  |  |  |  |  |  |  | 2.50 (1.02) | -.12 | .83 |  | 2.75 (0.77) | .32 | .77 |  | **<.001** | **<.001** | .030 |
| Exhibitionism (10) | 2.81 (1.03) | .62 | .17 | .43 | .77 | .07 | .35 | .05 | .03 | .17 |  |  |  |  |  |  |  |  |  |  |  | 2.54 (0.96) | .50 | .08 |  | 3.62 (0.8) | .38 | .20 |  | **<.001** | .054 | .113 |
| Authoritativeness (11) | 3.02 (1.14) | .67 | -.13 | .39 | .76 | -.36 | .14 | -.27 | .38 | -.25 | .43 |  |  |  |  |  |  |  |  |  |  | 2.72 (1.11) | .59 | -.26 |  | 3.92 (0.62) | .36 | -.12 |  | **<.001** | **<.001** | .060 |
| Thrill-Seeking (12) | 2.01 (1.04) | .70 | .19 | .69 | .47 | -.10 | .34 | -.08 | .28 | .15 | .39 | .34 |  |  |  |  |  |  |  |  |  | 1.64 (0.80) | .50 | .06 |  | 3.07 (0.94) | .38 | .20 |  | **<.001** | .054 | .065 |
| Grandiose Fantasies (13) | 2.38 (1.07) | .66 | .32 | .57 | .72 | .12 | .44 | .06 | .03 | .29 | .52 | .29 | .40 |  |  |  |  |  |  |  |  | 2.05 (0.97) | .49 | .27 |  | 3.35 (0.71) | .30 | .20 |  | **<.001** | .003 | .338 |
| Distrust (14) | 2.95 (1.00) | .24 | .66 | .51 | .07 | .19 | .40 | .25 | .08 | .34 | -.06 | .00 | .16 | .18 |  |  |  |  |  |  |  | 2.83 (1.06) | .11 | .68 |  | 3.31 (0.71) | .21 | .42 |  | **<.001** | .185 | **.000** |
| Manipulativeness (15) | 2.45 (1.08) | .77 | .18 | .72 | .60 | -.15 | .38 | -.10 | .30 | .05 | .46 | .57 | .49 | .42 | .22 |  |  |  |  |  |  | 2.07 (0.92) | .61 | .08 |  | 3.56 (0.67) | .57 | .09 |  | **<.001** | .185 | .896 |
| Exploitativeness (16) | 2.01 (1.10) | .79 | .33 | .85 | .51 | -.01 | .49 | .00 | .21 | .23 | .40 | .35 | .53 | .53 | .29 | .65 |  |  |  |  |  | 1.55 (0.73) | .51 | .26 |  | 3.35 (0.89) | .60 | .39 |  | **<.001** | .428 | .060 |
| Entitlement (17) | 1.89 (0.77) | .64 | .48 | .75 | .41 | .16 | .54 | .17 | .13 | .40 | .38 | .19 | .45 | .49 | .34 | .41 | .61 |  |  |  |  | 1.63 (0.60) | .34 | .44 |  | 2.64 (0.74) | .55 | .58 |  | **<.001** | .092 | .014 |
| Lack of Empathy (18) | 2.16 (0.99) | .62 | .19 | .72 | .23 | -.24 | .35 | -.12 | .46 | .03 | .10 | .22 | .45 | .26 | .32 | .41 | .58 | .45 |  |  |  | 1.85 (0.84) | .37 | .07 |  | 3.06 (0.87) | .47 | .26 |  | **<.001** | **.001** | .011 |
| Arrogance (19) | 2.07 (0.93) | .80 | .29 | .81 | .58 | -.06 | .48 | -.03 | .27 | .19 | .43 | .41 | .52 | .55 | .24 | .53 | .70 | .62 | .57 |  |  | 1.70 (0.63) | .57 | .15 |  | 3.18 (0.76) | .62 | .47 |  | **<.001** | .116 | **.000** |
| Acclaim-Seeking (20) | 3.31 (1.11) | .60 | -.03 | .33 | .78 | -.23 | .16 | -.16 | .25 | -.17 | .42 | .56 | .29 | .38 | .08 | .37 | .28 | .21 | .13 | .37 |  | 3.06 (1.13) | .55 | -.12 |  | 4.03 (0.66) | .36 | -.06 |  | **<.001** | .317 | .435 |

*Note*. Full sample: Correlations above *r* = .06 are significant at *p* < .05, correlations above *r* = .08 are significant at *p* < .01. Lower grandiosity subsample: Correlations above *r* = .08 are significant at *p* < .05, correlations above *r* = .10 are significant at *p* < .01. Higher grandiosity subsample: Correlations above *r* = .13 are significant at *p* < .05, correlations above *r* = .17 are significant at *p* < .01. The last three columns show significance of differences between means (*p* Δ *M*) and correlations (grandiosity [*p* Δ *r*1,x] and vulnerability [*p* Δ *r*2,x]). Significant differences after correction for multiple testing are printed in bold. Bonferroni correction was performed for the number of comparisons within each family of tests (i.e., comparisons within each column) for the last three columns.
